# In Silico and In Vitro Identification of 1,8-Dihydroxy-4,5-dinitroanthraquinone as a New Antibacterial Agent against *Staphylococcus aureus* and *Enterococcus faecalis*

**DOI:** 10.3390/molecules29010203

**Published:** 2023-12-29

**Authors:** Juliana Amorim, Viviana Vásquez, Andrea Cabrera, Maritza Martínez, Juan Carpio

**Affiliations:** Unidad de Salud y Bienestar, Facultad de Bioquímica y Farmacia, Universidad Católica de Cuenca, Av. Las Américas, Cuenca 010105, Ecuador

**Keywords:** antibacterial activity, nitrated anthraquinone, Gram-positive, pharmacophore, molecular docking, molecular dynamics

## Abstract

Increasing rates of bacterial resistance to antibiotics are a growing concern worldwide. The search for potential new antibiotics has included several natural products such as anthraquinones. However, comparatively less attention has been given to anthraquinones that exhibit functional groups that are uncommon in nature. In this work, 114 anthraquinones were evaluated using in silico methods to identify inhibitors of the enzyme phosphopantetheine adenylyltransferase (PPAT) of *Staphylococcus aureus*, *Enterococcus faecalis*, and *Escherichia coli*. Virtual screenings based on molecular docking and the pharmacophore model, molecular dynamics simulations, and free energy calculations pointed to 1,8-dihydroxy-4,5-dinitroanthraquinone (DHDNA) as the most promising inhibitor. In addition, these analyses highlighted the contribution of the nitro group to the affinity of this anthraquinone for the nucleotide-binding site of PPAT. Furthermore, DHDNA was active in vitro towards Gram-positive bacteria with minimum inhibitory concentration (MIC) values of 31.25 µg/mL for *S. aureus* and 62.5 µg/mL for *E. faecalis* against both antibiotic-resistant isolates and reference strains but was ineffective against *E. coli*. Experiments on kill-time kinetics indicated that, at the tested concentrations, DHDNA produced bacteriostatic effects on both Gram-positive bacteria. Overall, our results present DHDNA as a potential PPAT inhibitor, showing antibacterial activity against antibiotic-resistant isolates of *S. aureus* and *E. faecalis*, findings that point to nitro groups as key to explaining these results.

## 1. Introduction

The widespread threat of bacterial resistance leading to a decreased availability of therapeutic resources is a growing global concern [1]. The indiscriminate and unguided use of antibiotics in the treatment of bacterial infections has played a major role in accelerating this scenario [2,3]. Among the bacterial species exhibiting multidrug resistance are *Staphylococcus aureus*, *Enterococcus faecalis*, and *Escherichia coli* [4]. Although these bacteria are part of the normal human microbiota, under certain circumstances, they can cause a wide range of diseases. For this reason, there is a continuous search for new molecules with therapeutic potential [5]. The main mechanisms of bacterial resistance include mutations at the target sites that reduce affinity for the drug [6], the expression of efflux pumps that actively remove antibiotics from the cytoplasm [7], alterations in the membrane and cell wall that result in the reduced entry of antibiotics [8], and additionally, the expression of antibiotic-inactivating enzymes [9].

Phosphopantetheine adenylyltransferase (PPAT) is a key enzyme that, in bacteria, catalyzes the penultimate reaction in the synthesis of coenzyme A (CoA) [10]. This step consists of the transfer of an AMP moiety from ATP to 4′-phosphopantetheine, yielding dephospho-CoA and inorganic pyrophosphate. Due to the essential role of CoA in various cellular processes, such as amino acid metabolism and the biosynthesis of sterols, as well as the TCA cycle and fatty acid metabolism, this molecule is of central importance in bacterial metabolism [11,12,13]. Considering all these roles, the inhibition of this pathway is a very attractive strategy for antibacterial drug development [13]. Structurally, PPAT has three subsites, S1, S2, and S3, at the binding site, which accept and orient both substrates for catalysis. ATP binds at the S1 and S2 sites, while 4′-phosphopantetheine binds at S2 and S3. Previous studies have shown that some cycloalkyl pyrimidines inhibit the PPAT of *S. aureus*, acting as competitive and mixed inhibitors of 4′-phosphopantetheine and ATP, respectively [14].

Anthraquinones are a diverse group of molecules found mainly in plants and fungi but also present in some bacteria and insects [15]. Structurally, these molecules consist of three linearly fused six-membered rings resulting in a planar structure, with two ketone groups located at positions 9 and 10 of the central backbone ring [16]. Naturally occurring anthraquinones usually have functional groups such as hydroxyl, methyl, carboxyl, and methoxyl [17]. Apart from their use as natural colorants [18], anthraquinones have been continuously studied for their numerous biological effects. Among their properties are laxative [19], anti-inflammatory [20], anti-arthritic [21], anticancer [22], antiviral [23], and antifungal [24], as well as antibacterial potential [25,26,27]. The antibacterial effects of anthraquinones have been attributed to various mechanisms of action. These include the disruption of the bacterial cell membrane [28], inhibition of critical enzymes of bacterial metabolism [29], disturbance of cytokinesis, and alteration of DNA conformation [28,30]. In addition, due to their affinity for the nucleotide-binding sites (NBS) of dehydrogenases, kinases, and ATPases, some anthraquinones have been used in certain enzyme purification protocols [31].

To improve and expand the range of bioactivities of naturally occurring anthraquinones, efforts have been made for the synthesis of derivatives with functional groups not usually found in those extracted from natural sources. For instance, nitro derivatives such as 1,8-dihydroxy-4-nitro-anthraquinone have demonstrated a higher inhibitory activity against casein kinase-2 when compared to 1,8-dihydroxyanthraquinone [32]. Furthermore, the chlorinated derivative of naturally occurring emodin (3-methyl-1,6,8-trihydroxyanthraquinone) exhibits greater activity against some Gram-positive bacteria than the parent compound [28]. In the present study, we conducted in silico evaluations of 114 commercially available anthraquinones in search of potential PPAT inhibitors, followed by in vitro experiments to assess the antibacterial activity of the most promising anthraquinone against *S. aureus*, *E. faecalis*, and *E. coli*.

## 2. Results

### 2.1. Virtual Screenings Based on Molecular Docking and Pharmacophore Model

**Figure 1:**
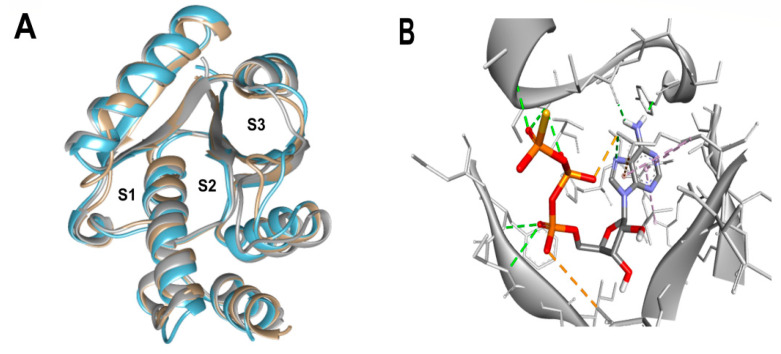
Three-dimensional representation of PPAT of *S. aureus*, *E. faecalis*, and *E. coli*, and 3D representation of *Sa*PPAT complexed with AGS. (**A**) Three-dimensional representation of the superposition of 4NAU (*Sa*PPAT, gray), 3ND6 (*Ef*PPAT, tan), and 6CCO (*Ec*PPAT, light blue) highlighting the subsites of interaction with ATP analog (S1 and S2), and with 4′-phosphopantetheine (S2 and S3). (**B**) Three-dimensional representation of 4NAU co-crystallized with AGS, highlighting the interactions at the NBS.

Based on the known ability of certain anthraquinones to bind to the nucleotide-binding site (NBS) of some enzymes [31] the potential of 114 molecules to inhibit PPAT was evaluated, given that it is a validated pharmacological target [14]. Figure 1A shows the comparison of the three-dimensional structures of the PPATs of *S. aureus* (*Sa*PPAT; PDB: 4NAU), *E. faecalis* (*Ef*PPAT; PDB: 3ND6), and *E. coli* (*Ec*PPAT; PDB: 6CCO) highlighting their different subsites. Importantly, in contrast to *Sa*PPAT and *Ef*PPAT, *Ec*PPAT was not co-crystallized with any ligand in the NBS. The virtual screening based on molecular docking identified 1,8-dihydroxy-4,5-dinitroanthraquinone (DHDNA) as the ligand with the best binding affinity for Gram-positive PPATs, (Supplementary Table S1). On the other hand, DHDNA ranked 52nd for *Ec*PPAT, despite having a quantitatively similar affinity to that for *Sa*PPAT and *Ef*PPAT. Interestingly, the derivative without nitro groups, 1,8-dihydroxyanthraquinone, exhibited a higher affinity for *Ec*PPAT, ranking 3rd, but, in complex with *Sa*PPAT and *Ef*PPAT, ranked 47th, and 78th, respectively. However, the derivative without hydroxyl groups, 1,8-dinitroanthraquinone, ranked 54th for *Sa*PPAT and 56th for both *Ef*PPAT and *Ec*PPAT. These results show that the presence of both the hydroxyl and nitro groups on the anthraquinone backbone is essential for the higher affinity of DHDNA for the PPAT of both Gram-positive bacteria, but for *Ec*PPAT, the hydroxyl groups are the main contributors to the affinity. Furthermore, when analyzing the effect of the substitution of hydroxyl groups with chlorine, exemplified by 1,8-dichloroanthraquinone, the affinity for PPAT of the three species was reduced, ranking 114th, 89th, and 47th for *Sa*PPAT, *Ef*PPAT, and *Ec*PPAT, respectively.

A possible explanation for the differences in screening results obtained using the PPAT of Gram-positive bacteria, compared to those obtained with the *E. coli* enzyme, may lie in the structure of its active sites as a consequence of the absence of a co-crystallized ligand at the NBS of *Ec*PPAT. Despite the high homology in NBS sequences across bacterial species, there are marked differences in the conformation of critical PPAT residues in the ATP-bound state compared to the unbound state [13].

Since molecular docking is very sensitive to even small variations in the structure of the active site [33], the dataset was evaluated using pharmacophore-based virtual screening as an orthogonal method to identify the best ligands. Due to the limited number of competitive inhibitors of PPAT identified, pharmacophoric features were inferred from the analysis of the interactions of the co-crystallized ATP analog (AGS) at the NBS of 4NAU, Figure 1B.

**Figure 2:**
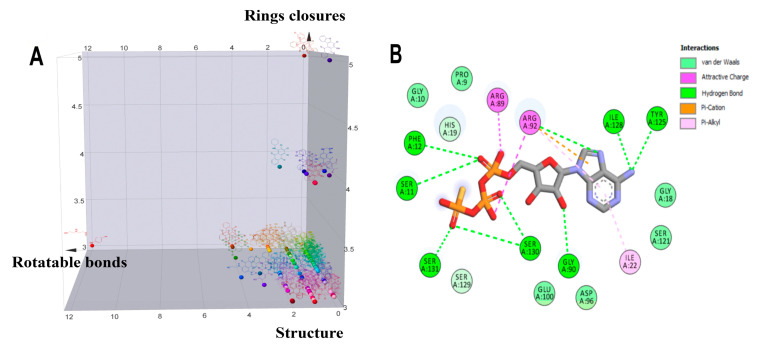
Analysis of the structural diversity of the database composed of 114 anthraquinones and a 2D interaction diagram of the complex 4NAU-AGS. (**A**) Three-dimensional distribution graph of the structures of 114 anthraquinones: x = number of rotatable bonds, y = number of ring closures, and z = chemical structure. (**B**) Two-dimensional diagram of the interaction of the complex 4NAU-AGS.

**Figure 3:**
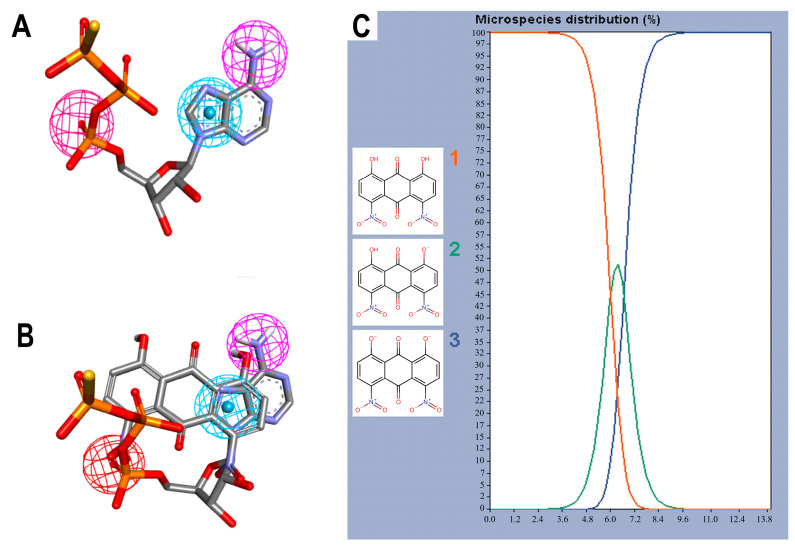
Pharmacophore features and species of 1,8-dihydroxy-4,5-dinitroanthraquinone (DHDNA) in the full pH range. (**A**) Features used to create the query pharmacophore model: hydrogen bond donor, aromatic system, and hydrogen bond acceptor are represented in magenta, blue and red spheres, respectively. (**B**) Overlap of AGS with DHDNA also represented in the same colors. These representations were generated with Discovery Studio using the coordinates obtained from Pharmit analyses. (**C**) Distribution of the three species of the DHDNA across the entire pH range, (1) protonated, (2) semi-protonated, and (3) deprotonated species.

In addition to this criterion, the structure of the molecules in the dataset was also taken into account [34], since, unlike AGS, anthraquinones are planar and most of the 114 molecules have between 0 and 2 rotational bonds, as well as only three rings, corresponding to their backbone, Figure 2A. The query pharmacophore model included: (1) hydrogen bond donor (amino group of adenine involved in hydrogen bonds with TYR125 and ILE128), (2) aromatic ring (imidazole forming pi-stacking interactions with ARG92), and (3) hydrogen bond acceptor (oxygen atom of alpha-phosphate involved in two hydrogen bonds with SER11 and PHE12), Figure 2B and Figure 3A.

The screening results show that, among the 114 anthraquinones, only 14 satisfied the pharmacophore model and had RMSD values < 0.8. At the same time, ATP included as the positive control had the best score (RMSD = 0.07), Supplementary Table S2. Notably, DHDNA once again ranked among the best ligands, reaching the second position (RMSD = 0.58). Furthermore, when exclusive shape constraints were set to a tolerance level of 0, DHDNA was the best ligand (RMSD = 0.43), indicating that its conformation in the active site matches the pharmacophore model without steric hindrance [35]. As shown in Figure 3B, one of its hydroxyls acts as a hydrogen bond donor, the anthraquinone core meets the pharmacophore requirement of the aromatic system, and the oxygen in the nitro group acts as a hydrogen bond acceptor. Taken together, these results support the hypothesis that DHDNA has the potential to target the NBS of PPAT.

Considering the strong effect of the nitro group on the electronic properties of organic molecules [32,36], it was decided to evaluate its influence on the protonation state of the two hydroxyl groups of the DHDNA structure. The results show that, at the cytoplasmic pH range from 7.2 to 7.6, the predominant species is the deprotonated (89–75.5%), followed by the semi-protonated (23–11%) and a small fraction of the protonated state (1.5–0.3%), Figure 3C. Based on these results, for the following analyses, the three states, i.e., protonated (pDHDNA), semi-protonated (sDHDNA), and deprotonated (dDHDNA), were included because, despite the lower abundance of sDHDNA and pDHDNA, their involvement in potential biological effects could not be ruled out.

**Figure 4:**
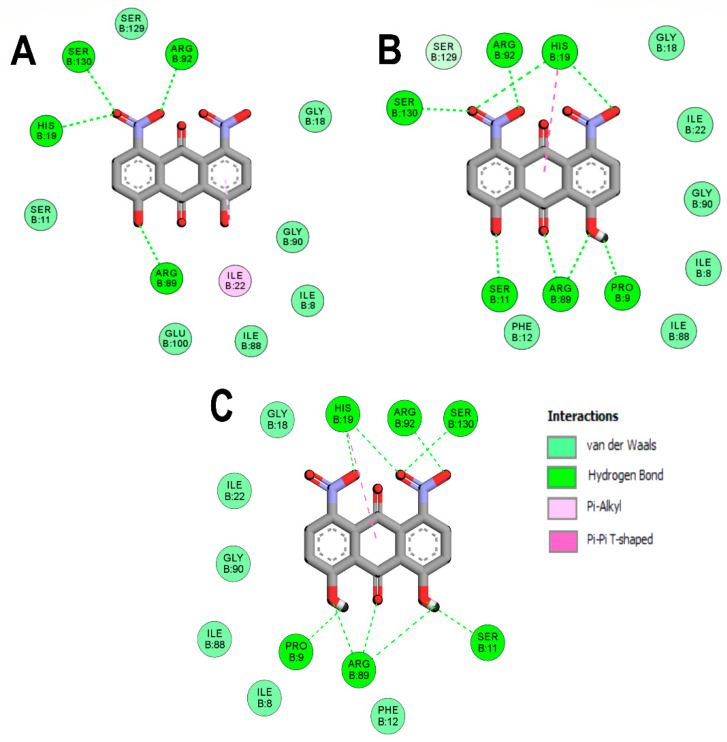
Analysis of the docked poses of the three species of DHDNA in a complex with 4NAU. (**A**) Two-dimensional interaction diagrams of 4NAU complexed with dDHDNA, (**B**) sDHDNA, and (**C**) pDHDNA.

To better understand the DHDNA interactions at the active site of the three PPATs, the interaction diagrams of the molecular docking results were analyzed. The results of the three species of DHDNA complexed with *Sa*PPAT (Figure 4A–C) show that their substituent groups (-OH, -O^−^, and -NO_2_) participated in interactions at the NBS, but dDHDNA established a lower number of hydrogen bonds. Notably, all the DHDNA states interact with critical residues, such as HYS19, which stabilizes ATP to nucleophilic attack at the α-phosphate group; ARG92, which is involved in the stabilization of the β-phosphate of the nucleotide; and the conserved SER130, which is part of a three-serine stretch located at the floor of subsite S1 [13].

**Figure 5:**
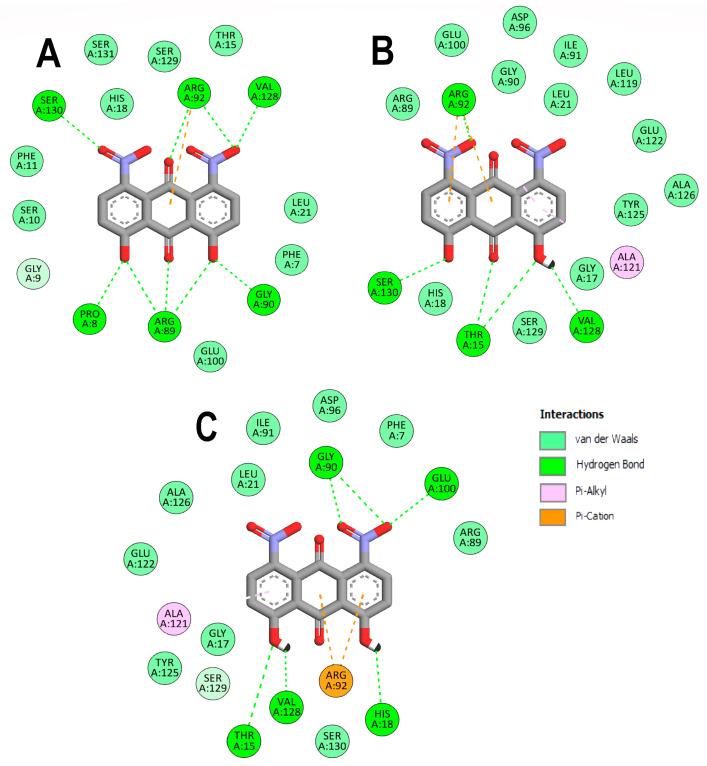
Analysis of the docked poses of the three states of DHDNA in complex with 3ND6. (**A**) Two-dimensional interaction diagrams of 3ND6 complexed with dDHDNA, (**B**) sDHDNA, and (**C**) pDHDNA.

On the other hand, the interactions of *Ef*PPAT with the three DHDNA species were less conserved, with only ARG92 and VAL128 interacting with all of them, Figure 5A–C. In addition, dDHDNA established more interactions with this target than with *Sa*PPAT, which is reflected in a binding affinity close to that of sDHDNA and pDHDNA. These results support the hypothesis that one of the main contributions of nitro groups to the binding affinities of DHDNA to these targets could be to favor the formation of interactions with polar amino acids in the active site.

**Figure 6:**
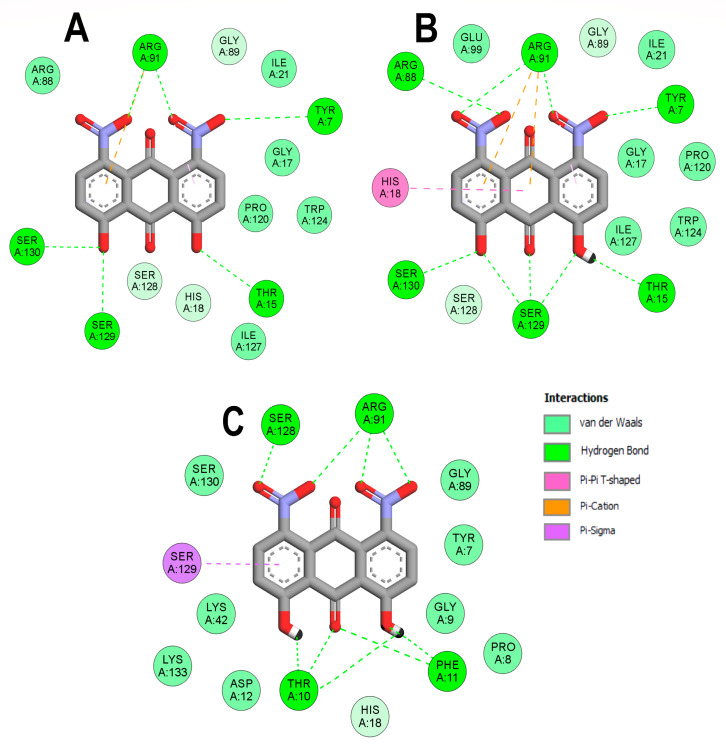
Analysis of the docked poses of the three states of DHDNA in complex with 6CCO. (**A**) Two-dimensional interaction diagrams of 6CCO complexed with dDHDNA, (**B**) sDHDNA, and (**C**) pDHDNA.

In the case of *Ec*PPAT, the only common hydrogen bond formed with the three DHDNA species involved ARG91, while TYR7, THR15, SER129, and SER130 were common to the complexes with the dDHDNA and sDHDNA states. At the same time, ARG88 only established bonds with the sDHDNA form, while THR10, PHE11, and SER128 interacted exclusively with the pDHDNA species, Figure 6A–C.

Considering that the affinities of the three species to the respective enzymes are close, collectively these results suggest that the effects of nitro groups on the deprotonation of hydroxyl groups, giving rise to negative charges, would not be the cause of the higher affinity of DHDNA for NBS compared to that of the other ligands analyzed.

### 2.2. Molecular Dynamic Simulations and Total Binding Free Energy Calculations

**Figure 7:**
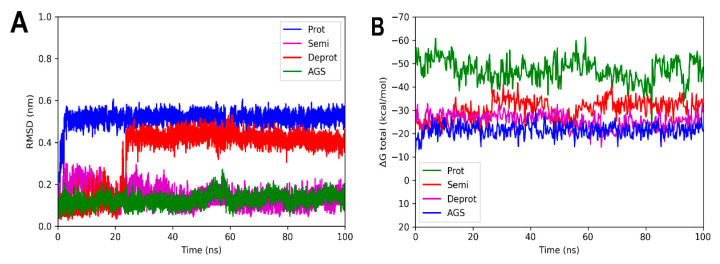
RMSD analyses and calculations of the binding free energy of 4NAU complexed with three states of DHDNA and with the co-crystallized ligand (AGS). (**A**) RMSD values. (**B**) Total ΔG energies.

**Figure 8:**
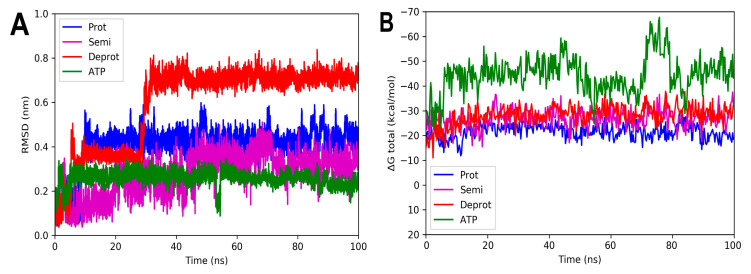
RMSD and free energy binding calculation analyses of 3ND6 complexed with three states of DHDNA and with the co-crystallized ligand (ATP). (**A**) RMSD values. (**B**) Total ΔG energies.

**Figure 9:**
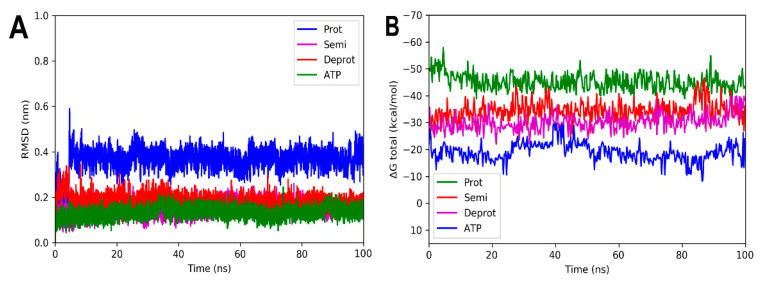
RMSD and free energy binding calculation analyses of 6CCO complexed with three states of DHDNA and with docked ATP. (**A**) RMSD values. (**B**) Total ΔG energies.

Molecular dynamic simulations were performed to evaluate the stability of the complexes formed between the three DHDNA species with each PPAT enzyme. Figure 7A shows that the profile of complexes with *Sa*PPAT was more stable compared to that with *Ef*PPAT (Figure 8A), but less stable compared to *Ec*PPAT (Figure 9A), throughout the time analyses. The complex with pDHDNA increased the RMSD during the first 2 ns until it reached 0.5 nm, a value maintained until the end of the run. At the same time, the *Sa*PPAT-dDHDNA complex showed, during the first 21 ns, an RMSD of a maximum of 0.25 nm, followed by an increase to a value of 0.4 nm, which was maintained until the end of the analysis time. In the case of the complex with sDHDNA, it presented an RMSD of less than 0.25 nm for most of the analysis time. Moreover, in the last 40 ns, this profile was similar to that of the co-crystallized ligand, which corroborates the higher stability of the complex with the semi-protonated state compared to those formed with the other species. Furthermore, as the negative values of the total binding energy calculated with MMPBSA suggest, the three DHDNA species, as well as the co-crystallized ligand (AGS), remain in the enzyme throughout the analysis time, Figure 7B.

On the other hand, the analyses of the complexes with *Ef*PPAT reveal RMSD values with greater fluctuations during most of the runs, Figure 8A. Among them, the complex with the semi-protonated form exhibited relatively better stability, showing an RMSD below 0.38 nm during most of the analysis time. However, despite fluctuations, even the less stable complex formed with the deprotonated state also did not leave the enzyme throughout the run, apparently, as suggested in its total binding energy plot (negative values), Figure 8B. It is noteworthy that, in the complexes with both enzymes of Gram-positive bacteria, there are no marked differences between the total binding energies, results that agree with the docking analyses.

For the complexes involving *Ec*PPAT and the three DHDHA states, the smallest variations in the RMSD values obtained were observed. The complex with the protonated form increased by 0.4 nm in the first 3 ns of the simulation, while the deprotonated and semi-protonated forms maintained an average of 0.18 and 0.17 nm, respectively, Figure 9A. In fact, the values of the latter two forms are very similar to those of the docked ATP used as control. In the same way as the other crystals, the total energy values remained practically constant throughout the simulation time, suggesting that the complexes remained integrated over time, Figure 9B.

### 2.3. Decomposition of Binding Free Energy Analysis

To further analyze the contributions of the different energies to the affinity of DHDNA for the three PPATs, binding energy decompositions were performed from calculations of the total binding free energy of the last 50 ns of each of the runs. The total ΔG for *Sa*PPAT complexed with pDHDNA, sDHDNA, and dDHDNA, were, respectively, −22.5, −24.3, and −31.0 kcal/mol (Supplementary Table S3). Additionally, among *Ef*PPAT and, respectively, pDHDNA, sDHDNA, and dDHDNA, the total ΔG were −21.1, −25.0, and −29.7 kcal/mol (Supplementary Table S4). At the same time, for the different states of DHDNA interacting with *Ec*PPAT, the authors obtained values of −21.8, −29.9, and −34.2 kcal/mol, respectively, for pDHDNA, sDHDNA, and dDHDNA (Supplementary Table S5).

In all the complexes studied, it was observed that the protonated state of DHDNA presents a higher contribution from Van der Waals interactions (ΔE, vdw), while the contributions from electrostatic interactions (ΔE, ele) are higher for the semi- and deprotonated forms. Although the latter component increases as a consequence of the deprotonation of one or two of the hydroxyl groups of DHDNA, respectively, the total energies of the three species are very close.

Furthermore, when analyzing the total ΔG of the complexes between the co-crystallized ligand of *Sa*PPAT (AGS), *Ef*PPAT (ATP), ATP docked to *Ec*PPAT, and the three DHDNA species, the values are markedly higher in the complexes involving nucleotides. This is an expected result, given that nucleotides not only occupy the pocket of adenosine at the NBS but also project at the site destined to interact with the phosphates of endogenous ATP. In fact, one possibility to further improve the binding energy of DHDNA with this enzyme would be to introduce modifications in its structure in an attempt to more closely resemble the interactions formed by phosphates of the endogenous ligand.

### 2.4. Pharmacokinetic and Target Fishing Predictions

Due to the limited information on biological assays with DHDNA, in silico analyses were conducted to obtain preliminary information on its pharmacokinetic profile and to identify potential targets in humans. As shown in Supplementary Table S6, predictions performed with SwissADME reveal that this anthraquinone has low gastrointestinal absorption, no potential to cross the blood–brain barrier (BBB), and could be a P-gp substrate. Considering these results, and in view of future in vivo assays, it may be necessary to develop a suitable formulation for the infection model selected for these experiments. In relation to metabolism, among the five cytochromes included in the predictive analyses, DHDNA would only be able to inhibit CYP2C9, which should be taken into account in possible future studies involving drugs metabolized by this enzyme.

Regarding the biological activity of DHDNA, it has been reported as a potent inhibitor of the dengue NS2B-NS3 viral protease [37] but, to our knowledge, there are no previous reports on their toxicological evaluation. Considering that anthraquinones from natural sources, such as rhein or emodin, can interact with human enzymes [16], target fishing analyses were conducted to identify possible human targets for DHDNA. The results in Supplementary Tables S7–S9 show that, compared to emodin and rhein, DHDNA has a lower probability of interacting with human targets, which may suggest a lower probability of causing off-target effects. Finally, the prediction of cytotoxicity to NIH/3T3 cells (mouse embryonic fibroblast) in Supplementary Table S10 indicates that DHDNA is not cytotoxic.

### 2.5. In Vitro Evaluation of the Antibacterial Activity of DHDNA

**Figure 10:**
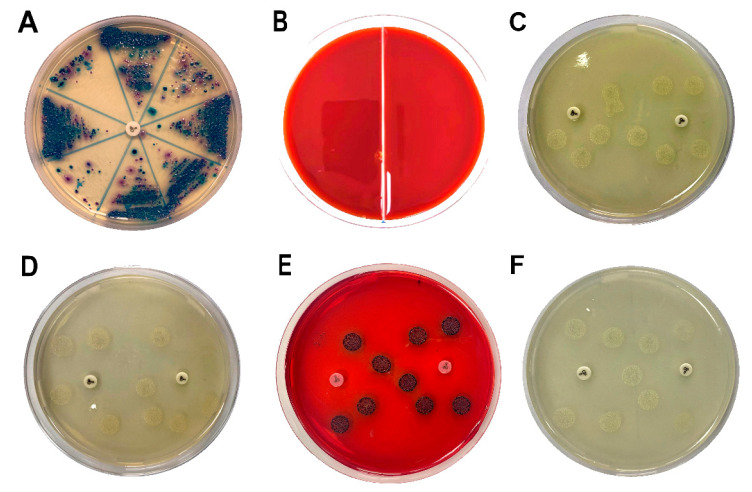
Samples streaked in chromogenic agar to isolate antibiotic-resistant *S. aureus*, *E. faecalis*, and *E. coli* and the effects of DHDNA on bacterial growth. (**A**) Chromogenic agar plate with ciprofloxacin disc (5 µg) to identify and isolate antibiotic-resistant bacteria. (**B**) *S. aureus* (left) and *E. faecalis* (right) exposed to 125 µg/mL of DHDNA. (**C**) *S. aureus* on agar plates with 1% DMSO in the proximity of ciprofloxacin discs to confirm antibiotic resistance. (**D**) *E. faecalis* in the same anterior conditions. (**E**) Colonies of *E. coli* exposed to 125 µg/mL of DHDNA and (**F**) on agar plates with 1% DMSO. DHDNA causes the red color of the agar plate. The images are representative of at least three independent experiments.

To evaluate the effect of DHDNA against *S. aureus*, *E. faecalis*, and *E. coli*, nine isolates, as well as reference strains, of each species were exposed to this compound using the agar macrodilution method. Since most of the bacteria we isolated showed resistance to ciprofloxacin, the nine isolates of each species we selected showed resistance to this antibiotic, Figure 10A. The results show that DHDNA at a concentration of 125 µg/mL completely inhibited the growth of *S. aureus* and *E. faecalis* (in both isolates and reference strains), Figure 10B, compared to the growth of both bacteria in the control medium for *S. aureus*, Figure 10C, and for *E. faecalis*, Figure 10D.

It is noteworthy that, when we exposed these bacteria to the anthraquinone without nitro groups, 1,8-dihydroxyanthraquinone, no growth inhibition was detected, demonstrating that the introduction of nitro groups is responsible for the antibacterial effect of DHDNA, Table 1. A previous study also reported the ineffectiveness of 1,8-dihydroxyanthraquinone against methicillin-resistant *S. aureus* [38]. Similarly, we observed that *S. aureus* and *E. faecalis* exposed to 1,8-dichloroanthraquinone did not show any differences concerning growth in the control medium, contrary to the enhanced antibacterial effect against *S. aureus* reported by the introduction of a chlorine atom in emodin [28].

Compared to the greater attention that *S. aureus* has attracted, relatively few studies have evaluated the effect of anthraquinones on *E. faecalis*. Among these few molecules tested, 1-(2-aminoethyl)piperazinyl-9,10-dioxo-anthraquinone [39] and emodin [28] have been reported to be inactive. In this context, the antibacterial effect exhibited by DHDNA is a relevant finding that highlights the contribution of nitro groups for the search and design of new anthraquinones active against Gram-positive bacteria. Although future in vitro experiments will be necessary to confirm the inhibition of PPAT by DHDNA, it is noteworthy that both the antibacterial activity of this compound, as well as the ineffectiveness of 1,8-dihydroxyanthraquinone and 1,8-dichloroanthraquinone, are in agreement with the in silico results.

On the other hand, when DHDNA was tested in the isolated and reference strains of *E. coli* under the same conditions mentioned above, no inhibitory effect was detected, Figure 10E. Interestingly, the bacteria exposed to DHDNA adopted a dark purple color, although they maintained the same morphology as the colonies in the control medium, Figure 10F. The results of the inactivity of DNDNA are in line with previous studies that have shown null or a reduced effect of several anthraquinones on Gram-negative bacteria. For example, the absence of antibacterial activity on *E. coli* was reported of anthraquinone (without functional groups) (100 µM), 1,5-dihydroxianthraquinone (10 µM), and 1,8-dihydroxianthraquinone (10 µM) [40]. In another recent study, anthraquinone mitoxantrone was up to 20 times less potent on Gram-negative bacteria compared to its effect on Gram-positive bacteria [41].

In addition, other authors, also, have shown that certain anthraquinones with hydroxyl, methoxyl, and carboxyl groups have low or no activity towards Gram-negative bacteria [42,43,44]. Among the causes of the reduced sensitivity of these bacteria to various antimicrobial agents are mechanisms that prevent or reduce their intracellular accumulation [45]. In fact, it has been proposed that the antibacterial effects of anthraquinones, such as emodin, could be explained mainly by their ability to cause direct damage to the bacterial membrane [30,46], or at least as a consequence of destabilizing it to allow them access to their intracellular targets [47]. In this context, the inactivity of DHDNA against *E. coli* could indicate that it does not exert such effects on the membrane, a hypothesis that will require future experiments to verify. On the other hand, structure–activity relationship studies have revealed that the presence of a primary amino group is a structural feature that favors the entry and retention of drugs in Gram-negative bacteria [48,49]. Considering that DHDNA does not include such a group, a lower intercellular accumulation of this compound could be an additional hypothesis for its lack of effect. Following this line of reasoning, the incorporation or substitution of some group of DHDNA by amino groups would allow the testing of this hypothesis and potentially expand its antibacterial spectrum.

### 2.6. Evaluation of the Potential of DHDNA to Resensitize Antibiotic-Resistant Bacteria

To obtain preliminary evidence of the potential of DHDNA to recover the effect of antibiotics against resistant bacteria, one isolate of each species exhibiting resistance to a greater number of antibiotics was selected among the samples used in previous experiments. The results in Table 2 show that the presence of DHDNA, at subinhibitory concentrations, did not sensitize any of the isolates to the effects of the antibiotics tested. A previous study showed that mitoxantrone synergizes vancomycin and other antibiotics such as ciprofloxacin against resistant *E. faecalis* strains. The synergism with vancomycin was related to the ability of this anthraquinone to induce oxidative stress and DNA damage [41]. Although it has been reported that their effects on the bacterial membrane could be another mechanism for anthraquinones to induce synergism [50], further studies will be needed to determine whether DHDNA does not sensitize the tested bacteria because it does not elicit such effects.

### 2.7. Determination of Minimum Inhibitory Concentrations

The next step was to determine the MIC of DHDNA in both reference and antibiotic-resistant Gram-positive strains. The MIC values for *S. aureus* and *E. faecalis* were, respectively, 31.125 µg/mL and 62.5 µg/mL, in both reference and isolate strains (Table 3). Comparing the activity of DHDNA with that of the previously reported chlorinated emodin [28], the latter has a higher activity against *S. aureus* (MIC = 4 µg/mL) but lower against *E. faecalis* (MIC = 256 µg/mL). In another study, Machado et al. tested 1,3-dimethoxy-8-hydroxy-6-methylanthraquinone (MIC > 32 µg/mL), 1,3-dimethoxy-2,8-dihydroxy-6-methylanthraquinone (MIC > 64 µg/mL), and 3-propyl-pyridinium anthraquinone derivative (MIC > 32 µg/mL) against both bacteria, without detecting antibacterial effects [51].

### 2.8. Time-Kill Kinetic Analysis

**Figure 11:**
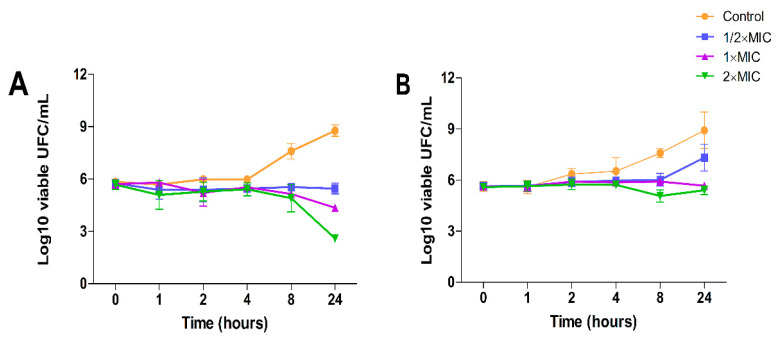
Time-kill kinetic assay using DHDNA at 1/2 × MIC, 1 × MIC, and 2 × MIC concentrations with exposure for 0, 1, 2, 4, 8, and 24 h. (**A**) Assay with reference strain of *S. aureus*. (**B**) Assay with reference strain of *E. faecalis*. The results are shown as the mean ± SD of at least three independent experiments.

The effects of DHDNA on the growth kinetics of both the *S. aureus* and *E. faecalis* reference strains were studied to determine whether its effect is bacteriostatic or bactericidal. Figure 11A shows that, from eight to 24 h of exposure at concentrations of 31.125 µg/mL (1 × MIC) and 62.5 µg/mL (2 × MIC), DHDNA induced a concentration-dependent reduction trend in the number of *S. aureus* relative to control. However, this reduction was ≤3 log10 cfu/mL relative to the number of bacteria in the initial inoculum. In *E. faecalis*, after 24 h of exposure, even to a concentration of 125 µg/mL of DHDNA (2 × MIC), the number of bacteria remains essentially unchanged compared to the initial count, Figure 11B.

These results suggest that DHDNA, at the tested concentrations, exerts a bacteriostatic effect in both *S. aureus* and *E. faecalis*. In line with this, a recent study reported that rhein (1,8-dihydroxyanthraquinone-3-carboxylic acid) also provoked bacteriostatic effects on *S. aureus* at concentrations of 12.5 µg/mL (1 × MIC) and 25 µg/mL (2 × MIC) [52].

It is important to note that, relative to growth on the control medium, exposure to DHDNA generated a decreasing trend in bacterial numbers only after eight hours of incubation. In this context, the delayed appearance of evidence of the effect of an agent on bacterial growth is often associated with molecules that act as inhibitors of cofactor biosynthesis [14]. Interestingly, considering that PPAT is involved in the synthesis of coenzyme A, these growth kinetic results coincide with those that would be expected in the case of inhibition of this enzyme by DHDNA.

## 3. Materials and Methods

### 3.1. Ligands and Targets Preparation for In Silico Analyses

The chemical structures of the anthraquinones and controls were downloaded from the ZINC20 database [53] in January 2023. Next, Marvin Sketch software was used to calculate the protonation state of the molecules to pH 7.4, and subsequently their 3D structures were generated with Avogadro-1.2 software [54]. Ligands were optimized by energy minimization using the MMFF94 force field with optimization of the steepest descent geometry with 500 steps, followed by the conjugate gradient algorithm with default parameters, and transformed into the MOL2 format. The analysis of the structural diversity of the anthraquinones in the database was carried out with DataWarrior V5.5.0 [55].

The affinities of selected anthraquinones for the ATP binding site of PPAT of *S. aureus*, *E. faecalis*, and *E. coli* (*Sa*PPAT, *Ef*PPAT, and *Ec*PPAT, respectively) were evaluated with molecular docking-based virtual screening. The 3D X-ray diffraction structures of *Sa*PPAT (PDB: 4NAU [14], chain B), *Ef*PPAT (PDB: 3ND6 [56], chain A), and *Ec*PPAT (PDB: 6CCO [57], chain A) were retrieved from the RCSB Protein Data Bank in January 2023. These targets were prepared using the Dock-prep module of UCSF-Chimera-1.16 [58] software applying the default parameters. The correct protonation state of certain amino acids, such as HIS-17, was inspected before docking analysis due to their critical role during catalysis [13]. Then, each structure was processed by the SPORES 1.3 tool using default parameters and saved in MOL2 format.

### 3.2. Molecular Docking Analyses

Molecular docking analyses were performed using the Protein-Ligand ANT System-1.2 software (PLANTS-1.2) [59]. All runs were performed with a radius of 12.5 Å, centering the coordinates on each co-crystallized ligand in the NBS, and by overlap with 4NAU for determination of this site at 6CCO. These coordinates of the *x*, *y*, and *z* axes were −15.5, 25.1, and 42.0 for 4NAU; −16.0, −11.0 and 31.0 for 3ND6; and −29.0, −42.0, and 51.0 for 6CCO. To ensure effective clustering, an RMSD value of 2.0 Å was established and default settings were used for all other parameters.

### 3.3. Pharmacophore-Based Virtual Screening

The pharmacophoric features were determined from the analysis of interactions of AGS, which is an ATP analog, co-crystallized with the A chain of 4NAU. The Pharmit server (https://pharmit.csb.pitt.edu/, (accessed on 20 November 2023)) [35] was used to perform the screening by applying the inferred features: hydrogen bond donor (x = −4.594, y = −40.148, and z = 18.476), aromatic system (x = −6.7402, y = −41.027, and z = 21.0482) from adenine moiety, and oxygen from the alpha-phosphate (x = −13.117, y = −42.054, and z = 21.638) as hydrogen bond acceptor. To perform the screening, the anthraquinone dataset was converted to SDF format and used as the input file to generate the respective conformers. The screenings with Pharmit were performed by applying: (1) no exclusive shape constraint, and (2) exclusive shape constraint with a tolerance level of 0.

### 3.4. Molecular Dynamic Simulations

All MD simulations of the complexes between the three states of DHDNA and each of the selected enzymes, as well as the complexes between the co-crystallized molecules, were performed using the software GROMACS-2021.4 [60], applying an all-atom CHARMM 36 force field [61]. The solvation water model employed for all systems was the water transferable intermolecular potential 3P (TIP3P), which was utilized within a periodically corrected cubic box, ensuring a minimum edge distance of 1.2 nm. To achieve system neutrality, Na^+^ and Cl^−^ ions were added. The steepest descent algorithm was then employed to perform 50,000 energy minimization steps to eliminate initial steric shocks.

The equilibration process consisted of two stages. Firstly, the system was equilibrated for 500 ps at a temperature of 310 K in the NVT ensemble. Subsequently, an equilibration period of 5000 ps was conducted in the NPT ensemble at a pressure of 1 bar. The production runs were carried out for a maximum duration of 100 ns, with the coordinates saved at regular intervals of 10 ps. To ensure accurate control of pressure and temperature, the leap-frog algorithm and Berendsen coupling were employed throughout the procedures [62]. The long-range electrostatic interactions were analyzed using the particle mesh Ewald (PME) algorithm [63], while the LINCS algorithm implementation regulated the covalent bonds [64].

### 3.5. Binding Free Energy Calculation

We carried out total binding free energy calculations from molecular dynamic trajectories to further investigate the magnitude and types of interactions that contribute to the total energy in these complexes. The MMPBSA methodology was employed [65], utilizing a single trajectory, and calculated using gmx-MMPBSA 1.5.7 software [66]. From the MD analyses, the results of all the runs were extracted in two different ways. The first considered the entire time of each run to extract the total energy, considering 500 snapshots. Subsequently, for the energy decomposition analyses, 500 snapshots were extracted from the last 50 ns of each MD run. The determination of free energies incorporated specific parameters: inp = 1, istrng = 0.15, and indi = 2. While these parameters were utilized, the remaining parameters adhered to the recommended settings of the software.

### 3.6. Pharmacokinetic, Target Fishing, and Cytotoxic Predictions

The pharmacokinetic predictions were carried out using the Swiss-ADME web server (http://www.swissadme.ch/, (accessed on 20 March 2023)) [67]. The predictions of potential human targets of the selected ligands (target fishing) were performed with the Swiss Target Prediction web server (http://www.swisstargetprediction.ch/, (accessed on 20 March 2023)) [68]. The results are presented as scores ranging from 0 to 1, where the value 1 corresponds to the most likely target of the query molecule. Prediction of cytotoxicity was performed using the MouseTox web server (http://www.swisstargetprediction.ch/, (accessed on 20 March 2023)) [69], which is a tool trained to predict cytotoxic compounds for NIH/3T3 cells. All these servers were accessed in January 2023.

### 3.7. Material

1,8-dihydroxy-4,5-dinitroanthraquinone (97%) (CAS 81-55-0), 1,8-dihydroxyanthraquinone (97%) (CAS: 117-10-2), 1,8-dichloroanthraquinone (97%) (CAS: 82-43-9), Mueller-Hinton agar, and Mueller-Hinton broth were purchased from Sigma-Aldrich (St. Louis, MO, USA). HiCrome™ UTI Agar was purchased from Hi-media Laboratory Ltd. (Mumbai, India). All antibiotic disks were purchased from Bioanalyse (Ankara, Turkey). Control strains of *S. aureus* (ATCC 25923), *E. faecalis* (ATCC 29212), and *E. coli* (ATCC 25922) were obtained from the American Type Culture Collection (Rockville, MD, USA). Dimethyl sulfoxide (DMSO) was obtained from Merck (Rahway, NJ, USA). All reagents used were of the highest grade available.

In the experiments, the maximum concentration of anthraquinones used was 125 µg/mL. Final concentrations in the culture media were obtained by using solutions of the anthraquinones prepared in DMSO and adding them to sterile Mueller-Hinton (MH) broth or molten MH agar. The final concentration of DMSO in the culture media used in all experiments was 1%.

### 3.8. Isolation and Identification of Bacteria

Nine isolates each of *S. aureus*, *E. faecalis*, and *E. coli* were obtained from contaminated surfaces or wastewater from animal farms, each isolate coming from different samplings. Samples were streaked on UTI chromogenic agar for the identification of characteristic colonies of each bacterium. In addition, since we decided to evaluate the anthraquinones against resistant bacteria, antibiotic disks were incorporated into the same agar immediately after streaking and incubated at 37 °C for 16 h. Representative colonies of each bacterium were picked from the proximity of the antibiotic discs, further subcultured on selective and differential media for each species, and stained with Gram stain reagents for microscopic examination. Finally, confirmed colonies were picked, cultured in broth, and subsequently stored with glycerol (15%) at −20 °C. Reference strains from *S. aureus*, *E. faecalis*, and *E. coli* were cultured and stored in the same manner as the isolates.

### 3.9. Antibacterial Activity and Minimal Inhibitory Concentration Assays

The bacterial susceptibility and determination of the MIC of the anthraquinones against each bacterium were conducted following the agar macrodilution method (Clinical and Laboratory Standards Institute guidelines). For these experiments, fresh colonies were streaked on MH agar for 16 h at 37 °C. Subsequently, colonies were suspended in saline, adjusting the cell density to 1 × 10^8^ cfu/mL. These suspensions were further diluted with a volume of saline sufficient to add on the agar surfaces (with anthraquinones and control media) a total of 25 µL of suspension containing 10^4^ cfu per spot. Lastly, after incubation, the number of colonies on the plates was counted. Anthraquinones that completely inhibited bacterial growth at a concentration of 125 µg/mL were considered active.

The determination of the MIC was carried out following the protocol mentioned above but using decreasing concentrations (125–15.625 µg/mL) of anthraquinone. The MIC value corresponds to the lowest concentration of the compound that completely inhibits the growth of the bacteria as detected by the unaided eye.

### 3.10. Evaluation of the Sensitizing Potential of DHDNA in Antibiotic-Resistant Bacteria

To gain preliminary insight into the potential of DHDNA to sensitize bacteria resistant to conventional antibiotics, the protocol described by Rangel et al. [70], with minor modifications, was used. One strain of each species (*S. aureus*, *E. faecalis*, and *E. coli*) was chosen from among the nine that were isolated and exposed to DHDNA dissolved in agar at the respective sub-MIC concentrations for each of these bacteria (15.625 µg/mL, 31.125 µg/mL, and 125 µg/mL). The bacteria selected were those that showed resistance to the greatest number of antibiotics in the sensitivity tests. Bacterial suspensions were prepared in saline to match the 0.5 McFarland turbidity standard and inoculated onto the agar surfaces using a sterile swab. Subsequently, antibiotic discs were incorporated, and the plates were incubated for 16 h at 37 °C. After incubation, the zones of inhibition around the discs on the control plates (with and without DMSO) were measured and compared with those on the plates with DHDNA.

### 3.11. Time-Kill Kinetic Assay

To identify whether the selected anthraquinone acts as a bacteriostatic or bactericidal agent, time-kill kinetic assays were performed. The procedure was as described by Huband et al. with minor modifications [71]. Experiments were performed using a log phase inoculum at a density of 1 × 10^6^ cfu/mL cultured at 37 °C. Reference strains of *S. aureus*, as well as *E. faecalis*, were exposed to concentrations equal to their respective 1/2 × MIC, 1 × MIC, and 2 × MIC. Aliquots of 100 μL were collected at 0, 1, 2, 4, 8, and 24 h of incubation and serially diluted in saline. Subsequently, 25 μL of each dilution was streaked on MHA plates and incubated for 16 h at 37 °C. To avoid the potential carry-over effect of the anthraquinone, the drops were allowed to dry before streaking them on the agar surfaces [72]. Finally, the number of colonies formed after incubation was counted on each plate. A compound is deemed bactericidal if it causes a reduction of ≥3 log10 in the number of colonies compared to the initial inoculum; conversely, when the reduction is ≤3 log10, the compound is classified as bacteriostatic [73].

All assays for antibacterial activity, MIC determination, sensitization potential assessment, and time-kill kinetics were performed at least three times as independent experiments.

### 3.12. Data Analysis and Visualizations

The 2D diagrams and 3D representations of the complexes were produced using the software Discovery Studio Visualizer-2021 and UCSF-Chimera-1.16. MD simulation analyses were visualized with GROMACS scripts in conjunction with Python scripts using the NumPy, Pandas, Matplotlib, Seaborn, and Pytraj libraries. The RMSD representations were generated from the alpha-carbon of the protein in the presence or absence of the ligands. Free energy binding calculations and time-kill kinetic assay were visualized using the NumPy, Pandas, and Matplotlib libraries.

## 4. Conclusions

In the search for new molecules showing antibacterial activity, naturally occurring anthraquinones have received more attention than synthetic derivatives. The in silico protocol we carried out to find unusual anthraquinones in nature with the potential to target the PPAT of *S. aureus*, *E. faecalis*, and *E. coli* identified 1,8-dihydroxy-4,5-dinitroanthraquinone (DHDNA) as a promising new inhibitor. In addition, these analyses suggested that nitro groups are critical, primarily for establishing interactions at the PPAT active site, increasing its affinity. In vitro experiments revealed that this compound exhibits a marked detrimental effect on the growth of both *S. aureus* and *E. faecalis* at lower concentrations than other reported anthraquinones and acts as a bacteriostatic agent. However, it was ineffective against *E. coli*, which could be associated with the known low permissiveness of Gram-negative bacteria to the entry of a wide range of antimicrobial agents. Furthermore, the lack of activity exhibited by 1,8-dihydroxyanthraquinone in both Gram-positive bacteria highlights the strong influence of nitro groups on the antibacterial effect of DHDNA. This is an important finding for the synthesis and evaluation of new nitro derivatives inspired by the anthraquinone structure. Collectively, the results of this work present DHDNA as a new antibacterial anthraquinone against *S. aureus* and *E. faecalis*, potentially acting as a PPAT inhibitor. Given that DHDNA demonstrated activity against antibiotic-resistant bacteria of both Gram-positive species and exhibited lower MIC values compared to several previously described anthraquinones, this compound deserves to be considered in future studies for further exploration of its antibacterial activity.

## Figures and Tables

**Table 1:** Growth of *S. aureus*, *E. faecalis*, and *E. coli* isolates and control strains in the presence of 1,8-dihydroxy-4,5-dinitroanthraquinone, 1,8-dihydroxyanthraquinone, and 1,8-dichloroanthraquinone at the concentration of 125 µg/mL.

Results of Growth of *S. aureus*, *E. faecalis*, and *E. coli* Exposed to Selected Anthraquinones.			
	*S. aureus*	*E. faecalis*	*E. coli*
1,8-dihydroxy-4,5-dinitroanthraquinone 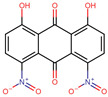	Absence	Absence	Presence
1,8-dihydroxyanthraquinone 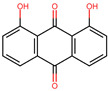	Presence	Presence	Presence
1,8-dichloroanthraquinone 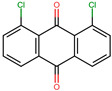	Presence	Presence	Presence

**Table 2:** Evaluation of the potential of DHDNA to resensitize resistant strains of *S. aureus*, *E. faecalis*, and *E. coli* against antibiotics. Selected colonies of *S. aureus*, *E. faecalis*, and *E. coli* were exposed to DHDNA dissolved in agar plates (15.5 µg/mL, 31.125 µg/mL, and 125 µg/mL, respectively) and incubated in the presence of antibiotic discs. The results presented were obtained from at least three independent experiments.

Effect of the Presence of Sub-MIC Concentrations of DHDNA in Bacteria Resistant to Selected Antibiotics	
*S. aureus*	
Ciprofloxacin (5 µg)	Resistant
Azithromycin (15 µg)	Resistant
Chloramphenicol (30 µg)	Resistant
Erythromycin (15 µg)	Resistant
Tetracycline (30 µg)	Resistant
Trimethoprim/sulfamethoxazole (25 µg)	Resistant
*E. faecalis*	
Ciprofloxacin (5 µg)	Resistant
Clindamycin (2 µg)	Resistant
Cefoxitin (30 µg)	Resistant
Cefuroxime (30 µg)	Resistant
Tetracycline (30 µg)	Resistant
Trimethoprim/sulfamethoxazole (25 µg)	Resistant
*E. coli*	
Ciprofloxacin (5 µg)	Resistant
Clarithromycin (15 µg)	Resistant
Ampicillin (10 µg)	Resistant
Amoxicillin (25 µg)	Resistant
Cephalexin (30 µg)	Resistant
Cefuroxime (30 µg)	Resistant
Chloramphenicol (30 µg)	Resistant
Trimethoprim/sulfamethoxazole (25 µg)	Resistant

**Table 3:** The minimal inhibitory concentration (MIC) of DHDNA on isolates and control strains of *S. aureus* and *E. faecalis*. The results were obtained from at least three independent experiments.

MIC (µg/mL)		
	*S. aureus*	*E. faecalis*
1,8-dihydroxy-4,5-dinitroanthraquinone	31.125	62.5

## Data Availability

All data generated or analyzed during this study are included in this published article and its Supplementary Information Files.

## Supplementary Materials

The following supporting information can be downloaded at https://www.mdpi.com/article/10.3390/molecules29010203/s1: Supplementary Table S1. Virtual screening results of 114 anthraquinones on the nucleotide-binding site of PPAT of *S. aureus*, *E. faecalis*, and *E. coli*. Highlighted in different colors are the molecules commented on in the main text; Supplementary Table S2. Results of pharmacophore-based virtual screening of 114 anthraquinones ranked by RMSD values. Analysis performed with Pharmit; Supplementary Table S3. Binding free energy calculation of SaPPAT-DHDNA states extracted from the single trajectory of MD simulation. The calculations were conducted on the final 50 ns run using gmx-MMPBSA-1.5.7 by analyzing 500 snapshots; Supplementary Table S4. Binding free energy calculation of EfPPAT-DHDNA states extracted from the single trajectory of MD simulation. The calculations were conducted on the final 50 ns run using gmx-MMPBSA-1.5.7 by analyzing 500 snapshots; Supplementary Table S5. Binding free energy calculation of EcPPAT-DHDNA states extracted from the single trajectory of MD simulation. The calculations were conducted on the final 50 ns run using gmx-MMPBSA-1.5.7 by analyzing 500 snapshots; Supplementary Table S6. Pharmacokinetic profile of DHDNA. Analysis was performed with SwissADME; Supplementary Table S7. Target prediction of DHDNA using Swiss Target Prediction; Supplementary Table S8. Target prediction of Emodin using Swiss Target Prediction; Supplementary Table S9. Target prediction of Rhein using Swiss Target Prediction; Supplementary Table S10. Cytotoxic activity of DHDNA on NIH/3T3 cells (mouse embryonic fibroblast).
